# Level Set Approach to Anisotropic Wet Etching of Silicon

**DOI:** 10.3390/s100504950

**Published:** 2010-05-17

**Authors:** Branislav Radjenović, Marija Radmilović-Radjenović, Miodrag Mitrić

**Affiliations:** 1 Institute of Physics, Pregrevica 118, 11080 Beograd, Serbia; E-Mail: marija@ipb.ac.rs; 2 Vinča Institute of Nuclear Sciences, P.O. Box 522, 11001 Beograd, Serbia; E-Mail: mmitric@vin.bg.ac.rs

**Keywords:** etching, level set, profile evolution, simulation

## Abstract

In this paper a methodology for the three dimensional (3D) modeling and simulation of the profile evolution during anisotropic wet etching of silicon based on the level set method is presented. Etching rate anisotropy in silicon is modeled taking into account full silicon symmetry properties, by means of the interpolation technique using experimentally obtained values for the etching rates along thirteen principal and high index directions in KOH solutions. The resulting level set equations are solved using an open source implementation of the sparse field method (ITK library, developed in medical image processing community), extended for the case of non-convex Hamiltonians. Simulation results for some interesting initial 3D shapes, as well as some more practical examples illustrating anisotropic etching simulation in the presence of masks (simple square aperture mask, convex corner undercutting and convex corner compensation, formation of suspended structures) are shown also. The obtained results show that level set method can be used as an effective tool for wet etching process modeling, and that is a viable alternative to the Cellular Automata method which now prevails in the simulations of the wet etching process.

## 1. Introduction

Micro- and Nano Electro Mechanical Systems (MEMS and NEMS) represent a rapidly expanding field of semiconductor fabrication technologies for producing micro and nano scale mechanical, electric, optical, fluidic, and other devices [1]. The inherently multi-physical and multi-disciplinary design of M(N)EMS devices demands new design methodologies including the integration of modeling, design, and simulation for M(N)EMS as early as possible in the course of the different life-cycle phases. In an ideal M(N)EMS design environment, it would be of great importance first to simulate the fabrication process steps in order to generate three dimensional (3D) geometrical models, including fabrication-dependent material properties and initial conditions. Refined control of etched profiles is one of the most important tasks of M(N)EMS manufacturing process. In spite of its wide use, the simulation of etching for M(N)EMS applications has been so far a partial success only, although a great number of commercial and academic research tools dedicated to this problem are developed.

Actually, two types of simulations exist [2]: the first category includes simulators describing etching process on the atomistic level, usually including the description of etched surface morphologies. The second type deals with the prediction of the etching profile evolution in engineering applications, typically including the combination of etching with other MEMS manufacturing techniques. The so called atomistic simulators based on cellular automata and Monte Carlo methods [2–7] belong to the former group. In this methods, a silicon substrate is represented by a large number of cells that reside in a crystalline lattice. During the etching process, the state of each individual cell, *i.e.*, whether it is removed from or remains within the lattice, is determined by the strength of chemical bounds and link status of its lattice neighbors. Also, the step-flow aspect [8] of wet etching process fits well into cellular automata method [7].

The most common type of the engineering simulators are so called geometrical simulators [9]. The etching profile is viewed as a set of planes propagating along their normal directions with the velocities obtained experimentally. The earlier geometric approaches implicitly assume that the etching is (quasi) equilibrium process that can be regarded as the reversal of crystal growth in saturated solutions, and that for every intermediate state Wulff theorem holds (a relatively new and mathematically rigorous exposition of the Wulff problem can be found in reference [10]). That means that the etched crystal is always in the minimum energy state with the shape determined by the Legendre transformation of the surface tension (Wulff shape). Within each time step, each plane forming a part of the surface evolves along its normal direction, experiencing a displacement according to the known etch rate for that particular plane. The change in geometry at the intersecting lines between adjacent crystallographic planes or at the edge of a masked region is determined using the Wulff-Jaccodine method [9], in order to avoid the problem of the missing values of the surface tensions. These simulators require the knowledge of complete etch rate diagrams, to be obtained somehow (experimentally, or in another calculations). Since only a finite set of etch rates can be obtained from experiment, it is particularly complicated to describe the evolution of curved non-crystallographic surfaces. Generally, continuum geometric representation of the etched surfaces is much more convenient for engineering applications, since it is usually necessary to integrate etching profiles evolution with other processes like chemical reactions, deposition, diffusion, viscous flow, *etc.* which are also described by continuum (partial differential) equations.

The level set method for evolving interfaces [11] belongs to the geometric type of methods, and it is specially designed for profiles which can develop sharp corners, change of topology and undergo orders of magnitude changes in speed. It is based on Hamilton-Jacobi type equation for the level set function using techniques developed for solving hyperbolic partial differential equations. This method is free on any implicit assumptions about the nature of the processes that force interface evolution, and the whole physics and chemistry of them are contained in just one parameter-normal component of the surface velocity. During last several years several variants of the level set methods have been developed with application to micro fabrication problems [12,13]. In this study we present an anisotropic etching simulator based on the sparse field method for solving the level set equations. The sparse-field method itself, developed by Whitaker [14], and broadly used in image processing community, is an alternative to the usual combination of narrow band and fast marching procedures for the computationally effective solving of the level set equations [15,16]. Our primary goal is to develop an accurate, stable and efficient 3D code for tracking of the etching profile evolution that includes different physical effects such as anisotropy and material-dependent propagation rates, yet being computationally effective to run on desktop PCs.

The paper is organized as follows: in Section 2 some aspect of the silicon wet etching process are discussed. After that, the relations describing the angular dependence of the etching rates, based on an interpolation procedure and silicon crystal symmetry properties, are derived. In Section 3 the necessary details for the implementation of the sparse field method for solving the level set equations in the case of etching rates defined in Section 2, are described. Section 4 contains simulation results for some interesting initial 3D shapes (cube and sphere), as well as some more practical examples illustrating anisotropic etching simulation in the presence of masks (simple square aperture mask, convex corner undercutting and convex corner compensation, formation of suspended structures).

## 2. Anisotropic Etching of Silicon: Angular Dependence of the Etching Rate

Although silicon etching techniques are currently undergoing a revolution driven by the incorporation of plasma etching process, anisotropic wet chemical etching is still the most widely used processing technique in silicon technology [1]. Not only the cost of wet etching systems is much lower than that of plasma types, but also certain features can only be realized using anisotropic wet etching. Very complicated 3D structures can be formed by this technique; it enables controlled undercutting of suspended structures, not possible by other microfabrication techniques. It is also referred to as “bulk micromachining”, since in this technology the body of the silicon wafer is etched away.

The anisotropy of the etching process is actually the orientation dependence of the etch rate. Regardless of the great amount of work done in this field, there is no generally accepted single theory for a mechanism that explains the great anisotropy in silicon wet etching. It is accepted [6] that the origin of this macroscopic anisotropy in the etching process lies in the crystal site-specificity of the etch rates at the atomistic level.

As stated earlier, in order to simulate the time evolution of 3D etching profiles it is essential that exact etch rates in all directions are known. In this paper we shall use the experimental values of etching rate for silicon in KOH solutions for three different etchant concentrations (30%, 40% and 50%) for etching temperature of 70 °C, and they are listed in Table 1. These values are taken from Reference [17], except the last row which originates from [18].

The etching rates for only limited number of directions are known, but they can be used to determine rate value in an arbitrary direction by an interpolation procedure. The problem of etching rate interpolation is equivalent to function interpolation over a sphere in 3D. For accuracy, the etching rate model must interpolate through the given etching rates and directions while maintaining its continuity, since possible requirement that the first derivative must be continuous also, is too high, as empirical studies have shown cusps in etching rate diagrams. Here we shall use etching rate model developed by Hubbard [9], that satisfies these conditions. Of course, this is not the only possibility; the problem of finding the optimal interpolation method is out of scope of this paper.

**Figure 1.**
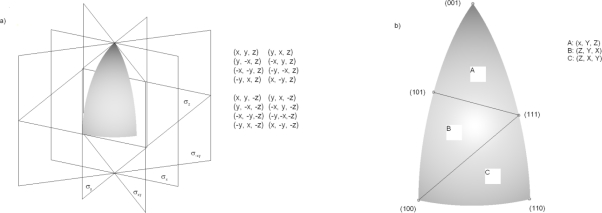
Etching rate interpolation region: (a) The angular section defined by the planes (0 ≤ *N_x_*; 0 ≤ *N_y_* ≤ *N_x_*; 0 ≤ *N_z_*). (b) Irreducible triangles (1/48th part of the all full angular extent). Curved triangle A is used as the interpolation region.

It is supposed that *x*, *y* and *z* axes are aligned with [100], [010] and [001] crystal directions, respectively. The point group of silicon's symmetry *m3m* (subgroup of Fd3m space group) contains 48 elements. Since it is not easy to assemble angular section using three principal directions with which the whole space can be covered by the symmetry operations, at first we shall use only 16 out of 48 symmetry elements for that purpose. As a result, we obtain only 1/16th of the all full angular extent (0 ≤ *θ* ≤ 90°; 0 ≤ ϕ ≤ 45°), or the wedge defined by the planes (0 ≤ *N_x_*; 0 ≤ *N_y_* ≤ *N_x_*; 0 ≤*N_z_*), as it is shown in Figure 1a, where the symmetry elements and the symmetry operations are denoted.

The second step is to divide this angular section into three equivalent (curved) triangles as it is shown in Figure 1b. These triangles are connected by the roto-inversion symmetry operations denoted in the same figure, with 3-fold roto-inversion axis going along (111) direction. The upper triangle, defined by (001), (101) and (111) directions, is the region where the etching rate should be interpolated. This is a 1/48th part of the all full angular extent, and in it for every arbitrary direction the equivalent point exists, which can be determined using enlisted symmetry operations.

**Figure 2.**
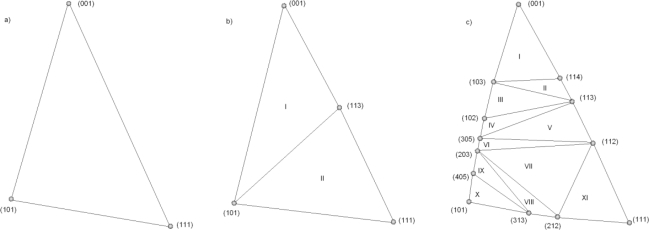
The (a) three- , (b) four- and (c) thirteen-parameters inetrpolation subregions.

The simplest method is to use only the experimental rate values for the principal directions [100], [110] and [111], since in three dimension three independent vectors are needed to define a basis. In that case, the interpolation region is shown in Figure 2a. Following the methodolgy described in Reference [9], the etching rate *R* in an arbitrary direction **N**′ is calculated in two steps. The first is to find the equivalent direction **N** (*N_x_*, *N_y_*, *N_z_*) in the interpolation region using symmetry operations; the second is to apply the appropriate interpolation relation:

(1) $$R(N\prime )=R(N)=\left[R_{100}(N_{z}-N_{x})+R_{110}(N_{x}-N_{y})+R_{111}N_{y}\right]/N_{z},$$

where *R*_hkl_ is etching rate in [hkl] direction. Details of this interpolation procedure can be found in [9]. Changing to the spherical coordinates:

(2) $$N_{x}=\text{sin}\theta \text{cos}\phi \;;\;N_{y}=\text{sin}\theta \text{sin}\phi \;;\;N_{z}=\text{cos}\theta$$

it is straightforward to obtain etching rate angular dependence.

**Figure 3.**
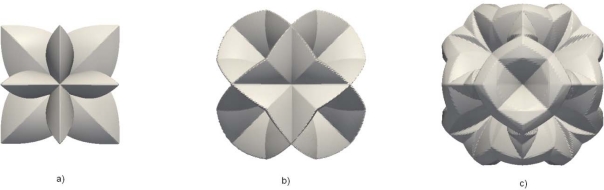
The angular dependence of the etching rate calculated using interpolation formulas with three (a), four (b) and thirteen (c) interpolation parameters (for 30% etchant concentration) listed in Table 1.

In Figure 3a the resulting three-parameter etching rate is shown in the full angular extent. The presence of cusps in the etching rates implies the existence of facets in the etching profiles. Better interpolation results can be obtained if additional experimentally obtained rates are included. For example, if include the first most important (high index) planes {311}, the interpolation region will consist of two sections: I [101, 001, 113], and II [101, 113, 111], as it is depicted in Figure 2b. Then, the etching rate *R* in an arbitrary direction **N** (*N_x_*, *N_y_*, *N_z_*) will be given by the relation:

(3) $$R(N)=\{\begin{array}{c} \left[R_{100}(N_{z}-N_{x}-{2N}_{y})+R_{110}(N_{x}-N_{y})+{3R}_{311}N_{y}\right]/N_{z};\;N\in I\\ \left[R_{111}\left(N_{x}+{2N}_{y}-N_{z}\right)/2+R_{110}(N_{x}-N_{y})+{3R}_{311}(N_{z}-N_{x})/2\right]/N_{z};\;N\in \text{II} \end{array}$$

Figure 3b shows the four-parameter angular dependence of the etching rate. There is no any principal difficulty in including other known high index etching rates, only the resulting analytical expression will become more complicated. In our previous papers [19], [20] we have used only these interpolation formula. Also, the interpolation procedure has been performed on the whole wedge, shown in Figure 1. as it was suggested initially in [9]. Here we shall write expression obtained using etching rates in thirteen directions, listed in Table 1, in order to improve our simulation model. Actually, these all that we could find in literature, although it is possible to find more data in commercial sources. In that case, the interpolation region is divided in eleven subregions, as it is shown in Figure 2c. The resulting etching rate angular dependence is shown in Figure 3c. It is obvious that it is important to include as many as possible parameters (experimental values of the etching rates) in order to increase precision of the interpolation procedure.

(4) $$R(N)=\{\begin{array}{c} \left[R_{100}(N_{z}-{3N}_{x}-N_{y})+{3R}_{310}(N_{x}-N_{y})+{4R}_{411}N_{y}\right]/N_{z};\;\;N\in I\\ \left[{3R}_{310}(N_{x}-N_{y})+{3R}_{311}(3N_{x}+N_{y}-N_{z})+{4R}_{411}(N_{z}-{3N}_{x})\right]/N_{z};\;\;N\in \text{II}\\ \left[{3R}_{310}(N_{z}-{2N}_{x}-N_{y})+{2R}_{210}({3N}_{x}-N_{z})+{3R}_{311}N_{y}\right]/N_{z};\;\;N\in \text{III}\\ \left[{2R}_{210}({3N}_{z}-{5N}_{x}-{4N}_{y})+{5R}_{530}({2N}_{x}-N_{y}-N_{z})+{3R}_{311}N_{y}\right]/N_{z};\;\;N\in \text{IV}\\ \left[{5R}_{530}(N_{x}-N_{y})/3+{2R}_{211}({5N}_{x}+{4N}_{y}-3N_{z})/3+R_{311}({3N}_{z}-5N_{z}-N_{y})\right]/N_{z};\;\;N\in V\\ \left[{5R}_{530}({2N}_{z}-{3N}_{x}-N_{y})+{3R}_{320}({5N}_{x}+N_{y}-{3N}_{z})+{2R}_{211}N_{y}\right]/N_{z};\;\;N\in \text{VI}\\ \left[R_{320}(N_{z}-{2N}_{y})+{2R}_{221}({3N}_{x}+N_{y}-{2N}_{z})+{2R}_{211}({2N}_{z}+{2N}_{y}-{3N}_{x})/3\right]/N_{z};\;\;N\in \text{VII}\\ \left[{3R}_{320}(N_{z}-N_{x})+{3R}_{331}({3N}_{x}-{2N}_{y}-{2N}_{z})+{2R}_{221}({2N}_{z}-{3N}_{x}+{3N}_{y})\right]/N_{z};\;\;N\in \text{VIII}\\ \left[{3R}_{320}({4N}_{z}-5N_{x}+{3N}_{y})/2+{5R}_{540}({3N}_{x}-{3N}_{y}-{2N}_{z})/2+{3R}_{331}N_{y}\right]/N_{z};\;\;N\in \text{IX}\\ \left[{5R}_{540}(N_{z}-N_{x})+R_{110}({5N}_{x}-{3N}_{y}-{4N}_{z})+{3R}_{331}N_{y}\right]/N_{z};\;\;N\in X\\ \left[{2R}_{211}(N_{z}-N_{x})+{2R}_{221}(N_{z}-N_{y})+R_{111}({2N}_{y}-N_{z})\right]/N_{z};\;\;N\in \text{XI} \end{array}$$

**Figure 4.**
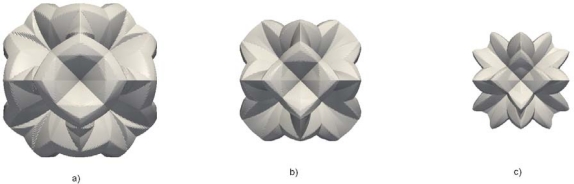
The angular dependence of the etching rate calculated using interpolation formulas with thirteen-parameters for three etchant concentrations: (a) 30%, (b) 40% and (c) 50%.

In Figure 4 the angular dependencies for three different etchant concentrations are shown. As can be seen, the increase of concentration slows down etching process, but the shapes of these functions are (roughly) identical.

It is important to remember that all physical aspects of the etching process are contained in these angular dependences, and that they determine time evolution of the feature profile completely, appearance and disapearrance of particular planes and the final profile. For different values of the parameters these shapes look different. Inclusion of additional planes will also change the shape of angular dependences.

## 3. Level Set Method for Non-Convex Hamiltonians

Level set method, introduced by Osher and Sethian [11], is a powerful technique for analyzing and computing moving fronts in a variety of different settings. The level sets are used in image processing, computer vision, computational fluid dynamics, material science, and many other fields. Detailed exposition of the theoretical and numerical aspects of the method, and applications to different areas can be found in books [15,16], and recent review articles [21,22]. The basic idea behind the level set method is to represent the surface in question at a certain time *t* as the zero level set (with respect to the space variables) of a certain function *φ* (*t*, **x**), the so called level set function. The initial surface is given by {**x** | *φ* (0, **x**) = 0}. The evolution of the surface in time is caused by “forces” or fluxes of particles reaching the surface in the case of the etching process. The velocity of the point on the surface normal to the surface will be denoted by *R* (*t*, **x**), and is called velocity function. For the points on the surface this function is determined by physical and chemical models of the ongoing processes. The velocity function generally depends on the time and space variables and we assume that it is defined on the whole simulation domain. At a later time *t* > 0, the surface is as well the zero level set of the function *φ* (*t*, **x**), namely it can be defined as a set of points {**x**∈ℜ^n^ | *φ* (*t*, **x**) = 0}. This leads to the level set equation:

(5) $$\frac{\partial \varphi }{\partial t}+\;R(t,x)\;\left|\nabla \varphi \right|\;=\;0$$

in the unknown function *φ* (*t*, **x**), where *φ* (0, **x**) = 0 determines the initial surface. Having solved this equation the zero level set of the solution is the sought surface at all later times. Actually, this equation relates the time change to the gradient via the velocity function. In the numerical implementation the level set function is represented by its values on grid nodes, and the current surface must be extracted from this grid. In order to apply the level set method a suitable initial function *φ* (0, **x**) has to be defined first. The natural choice for the initialization is the signed distance function of a point from the given surface. As already stated, the values of the velocity function are determined by the physical models.

The equation (5) can be rewritten in Hamilton-Jacobi form:

(6) $$\frac{\partial \varphi }{\partial t}+H(\nabla \varphi \;(t,x))=0,$$

where the Hamiltonian is given by *H* = *R* (*t*, **x**)|∇*φ*(*t*, **x**)| (in this context the term “Hamiltonian” denotes a Hamiltonian function, not an operator). A detailed exposition about the Hamilton-Jacobi equation, the existence and uniqueness of its solution (especially about its viscosity solutions), can be found in Reference [23]. We say that such a Hamiltonian is convex (in ℜ*^n^*) if the following condition is fulfilled:

(7) $$\frac{\partial^{2}H}{\partial \varphi_{x_{i}}\partial \varphi_{x_{j}}}\geq 0,$$

where *φ_x_i__* is a partial derivative of *φ*(*t*, **x**) with respect of *x_i_*. If the surface velocity *R* (*t*, **x**) does not depend on the level set function *φ*(*t*, **x**) itself, this condition is usually satisfied. In that case, we can say that the problem is of free boundary type. In that case the spatial derivatives of *φ* can be approximated using the Engquist-Osher upwind finite difference scheme, or by ENO (higher-order essentially non-oscillatory) and WENO (weighted essentially non-oscillatory) discretization schemes, that requires the values of this function at the all grid points considered. The resulting semi-discrete equations can be solved using explicit Euler method, or more precisely by TVD (total-variation diminishing) Runge-Kutta time integration procedure (see Reference [15] and [16] for the details).

Several approaches for solving level set equations exist which increase accuracy while decreasing computational effort. They are all based on using some sort of adaptive schemes. The most important are narrow band level set method, widely used in etching process modeling tools, and recently developed sparse-field method [14], implemented in medical image processing ITK library [24]. The sparse-field method use an approximation to the distance function that makes it feasible to recompute the neighborhood of the zero level set at each time step. It computes updates on a band of grid points that is only one point wide. The width of the neighborhood is such that derivatives for the next time step can be calculated. This approach has several advantages. The algorithm does precisely the number of calculation needed to compute the next position of the zero level set surface. The number of points being computed is so small that it is feasible to use a linked-list to keep a track of them, so at each iteration only those points are visited whose values control the position of the zero level set surface. As a result, the number of computations increases with the size of the surface, rather than with the resolution of the grid.

The non-convex Hamiltonians are characteristic for anisotropic etching and deposition simulations [13].The upwind finite difference scheme cannot be used in the case of non-convex Hamiltonians. The simplest scheme that can be applied in these cases is the Lax-Friedrichs, one which relies on the central difference approximation to the numerical flux function, and preserves monotonicity through a second-order linear smoothing term [16]:

(8) $$\begin{array}{l} \varphi_{\mathrm{ijk}}^{n+1}=\varphi_{\mathrm{ijk}}^{n}-\Delta t[H\left(\frac{D_{\mathrm{ijk}}^{-x}+D_{\mathrm{ijk}}^{+x}}{2},\frac{D_{\mathrm{ijk}}^{-y}+D_{\mathrm{ijk}}^{+y}}{2},\frac{D_{\mathrm{ijk}}^{-z}+D_{\mathrm{ijk}}^{+z}}{2}\right)\\ -\frac{1}{2}\alpha_{x}\left(D_{\mathrm{ijk}}^{+x}-D_{\mathrm{ijk}}^{-x}\right)-\frac{1}{2}\alpha_{y}\left(D_{\mathrm{ijk}}^{+y}-D_{\mathrm{ijk}}^{-y}\right)-\frac{1}{2}\alpha_{z}\left(D_{\mathrm{ijk}}^{+z}-D_{\mathrm{ijk}}^{-z}\right)] \end{array}$$

where $D_{\mathrm{ijk}}^{+x(y,z)}$ and $D_{\mathrm{ijk}}^{-x(y,z)}$ are usual forward and backward differences:

(9) $$\begin{array}{l} D_{\mathrm{ijk}}^{+x}=\frac{\varphi_{i+1,j,k}^{n}-\varphi_{i,j,k}^{n}}{\Delta x},\;D_{\mathrm{ijk}}^{-x}=\frac{\varphi_{i,j,k}^{n}-\varphi_{i-1,j,k}^{n}}{\Delta x}\\ D_{\mathrm{ijk}}^{+y}=\frac{\varphi_{i,j+1,k}^{n}-\varphi_{i,j,k}^{n}}{\Delta y},\;D_{\mathrm{ijk}}^{-y}=\frac{\varphi_{i,j,k}^{n}-\varphi_{i,j-1,k}^{n}}{\Delta y}\\ D_{\mathrm{ijk}}^{+z}=\frac{\varphi_{i,j,k}^{n}-\varphi_{i,j,k+1}^{n}}{\Delta z},\;D_{\mathrm{ijk}}^{-z}=\frac{\varphi_{i,j,k}^{n}-\varphi_{i,j,k-1}^{n}}{\Delta z} \end{array}$$

and *α_x_* (*α_y_*, *α_z_*) is a bound on the partial derivative of the Hamiltonian with respect to the first (second, third) argument:

(10) $$\alpha_{x}=\text{max}\left|\frac{\partial H}{{\partial \varphi }_{x}}\right|,\;\alpha_{y}=\text{max}\left|\frac{\partial H}{{\partial \varphi }_{y}}\right|,\;\alpha_{z}=\text{max}\left|\frac{\partial H}{{\partial \varphi }_{z}}\right|$$

The terms on the second row of the above equation are the smoothing terms. They are similar to the second derivatives in each variable. In general, these terms need not be calculated exactly. Overestimated values will produce non-realistic smoothing of the sharp corners in the implicit surfaces. Too little smoothing usually leads to numerical instabilities in calculations. In Reference [25] it is shown that it is possible to use the Lax-Friedrichs scheme in conjunction with the sparse field method, and to preserve sharp interfaces and corners by optimizing the amount of smoothing in it.

It is essential to express the etching rates in terms of the level set function itself in order to obtain level set equation in Hamilton-Jacobi form. To accomplish this goal, we start from the facts that the unit vector normal to the zero level set is given by **N** = ∇*φ*/ |∇*φ*|, and that the angles *θ* and *ϕ* are connected to the level set function by the $\text{sin}\vartheta =\sqrt{1-\varphi_{z}^{2}/{\left|\nabla \varphi \right|}^{2}}$ and *tgφ* = *φ_y_*/ |∇*φ*|. In this way the rate *R* can be expressed in terms of the geometrical properties of the level set function itself, and the Hamiltonian becomes:

(11) $$H(\nabla \varphi )=\left|\nabla \varphi \right|\left[(R_{110}-R_{100})\varphi_{x}+(R_{111}-R_{110})\varphi_{y}+R_{100}\varphi_{z}\right]/\varphi_{z}$$

for the simple etching rate angular dependence given by the relation (1). In order to implement Lax-Friedrichs scheme it is necessary to find first derivatives appearing in (10). After some straightforward algebraic manipulations the following relations can be obtained:

(12) $$\frac{\partial H}{{\partial \varphi }_{x}}=\left\{R_{111}\varphi_{x}\varphi_{y}+(R_{110}-R_{100})\left({\left|\nabla \varphi \right|}^{2}+\varphi_{x}^{2}\right)+\left(R_{110}\varphi_{y}-R_{100}\varphi_{z}\right)\varphi_{x}\right\}/\left(\varphi_{z}\left|\nabla \varphi \right|\right)$$

(13) $$\frac{\partial H}{{\partial \varphi }_{y}}=\left[R_{100}\varphi_{y}(\varphi_{z}-\varphi_{x})+(R_{111}-R_{110})\left({\left|\nabla \varphi \right|}^{2}+\varphi_{y}^{2}\right)+R_{110}\varphi_{y}\varphi_{x}\right]/\left(\varphi_{z}\left|\nabla \varphi \right|\right)$$

(14) $$\frac{\partial H}{{\partial \varphi }_{z}}=\left\{\left[(R_{100}-R_{110})\varphi_{x}+(R_{110}-R_{111})\varphi_{y}\right](\varphi_{x}^{2}+\varphi_{y}^{2})+R_{100}\varphi_{z}^{3}\right\}/\left(\varphi_{z}^{2}\left|\nabla \varphi \right|\right)$$

If the high index planes {311} are included, the expressions become more complicated because the interpolation region is then divided in two subregions. Since one of the goals of this study is investigate the influence of these planes on the final outcomes, we shall write them explicitly. The Hamiltonian corresponding to the relation (3) has the form:

(15) $$H(\nabla \varphi )=\{\begin{array}{c} \left|\nabla \varphi \right|\left[(R_{110}-R_{100})\varphi_{x}+({3R}_{311}-{2R}_{100}-R_{110})\varphi_{y}+R_{100}\varphi_{z}\right]/\varphi_{z};\;\nabla \varphi \in I\\ \left|\nabla \varphi \right|\left[({3R}_{311}/2+R_{110}+R_{111}/2)\varphi_{x}+(R_{111}-R_{110})\varphi_{y}+({3R}_{311}-R_{111})\varphi_{z}/2\right]/\varphi_{z};\;\nabla \varphi \in \text{II} \end{array}$$

while the derivatives are:

(16) $$\frac{\partial H}{{\partial \varphi }_{x}}=\{\begin{array}{c} \left\{{3R}_{311}\varphi_{x}\varphi_{y}+(R_{110}-R_{100}){\left|\nabla \varphi \right|}^{2}+\varphi_{x}\left[(R_{110}-R_{100})\varphi_{x}-(R_{110}+{2R}_{100})\varphi_{y}+R_{100}\varphi_{z}\right]\right\}/\left(\varphi_{z}\left|\nabla \varphi \right|\right);\;\nabla \varphi \in I\;\\ \left\{({2R}_{110}+R_{111}-{3R}_{311}){\left|\nabla \varphi \right|}^{2}+\varphi_{x}\left[{2R}_{110}(\varphi_{x}-\varphi_{y})+R_{111}(\varphi_{x}+{2\varphi }_{y}-\varphi_{z})+{3R}_{311}(\varphi_{z}-\varphi_{x})\right]\right\}/\left({2\varphi }_{z}\left|\nabla \varphi \right|\right);\;\nabla \varphi \in \text{II} \end{array}$$

(17) $$\frac{\partial H}{{\partial \varphi }_{y}}=\{\begin{array}{c} \left\{\left({3R}_{311}-{2R}_{100}-R_{110}\right){\left|\nabla \varphi \right|}^{2}+\varphi_{y}\left[R_{110}\left(\varphi_{x}-\varphi_{y}\right)+{3R}_{311}\varphi_{y}+R_{100}\left(\varphi_{z}-\varphi_{x}-{2\varphi }_{y}\right)\right]\right\}/\left(\varphi_{z}\left|\nabla \varphi \right|\right);\;\nabla \varphi \in I\\ \left\{\left(R_{111}-R_{110}\right){\left|\nabla \varphi \right|}^{2}+\varphi_{y}\left\{R_{110}\left(\varphi_{x}-\varphi_{y}\right)+\left[R_{111}\left(\varphi_{x}+2\varphi_{y}-\varphi_{z}\right)+{3R}_{311}\left(\varphi_{z}-\varphi_{x}\right)\right]/2\right\}\right\}/\left(\varphi_{z}\left|\nabla \varphi \right|\right);\;\nabla \varphi \in \text{II} \end{array}$$

(18) $$\frac{\partial H}{\partial \varphi_{z}}=\{\begin{array}{c} \left\{\left[\left(R_{100}-R_{110}\right)\varphi_{x}+\left({2R}_{100}+R_{110}-{3R}_{311}\right)\varphi_{y}\right]\left(\varphi_{x}^{2}+\varphi_{y}^{2}\right)+R_{100}\varphi_{z}^{3}\right\}/\left(\varphi_{z}^{2}\left|\nabla \varphi \right|\right);\;\nabla \varphi \in I\\ \left\{\left[\left({3R}_{311}-{2R}_{110}-R_{111}\right)\varphi_{x}+2\left(R_{110}-R_{111}\right)\varphi_{y}\right]\left(\varphi_{x}^{2}+\varphi_{y}^{2}\right)+\left({3R}_{311}-R_{111}\right)\varphi_{z}^{3}\right\}/\left({2\varphi }_{z}^{2}\left|\nabla \varphi \right|\right);\;\nabla \varphi \in \text{II} \end{array}$$

The same procedure can be used to derive relations when thirteen experimental values of the etching rates for interpolation are used (eleven subregions, Figure 2c). Namely, using thirteen-parameters formula (4) the following Hamiltonian has been obtained:

(19) $$H(\nabla \varphi )=\{\begin{array}{c} \left|\nabla \varphi \right|\left[3(R_{310}-R_{100})\varphi_{x}+({4R}_{411}-R_{100}-{3R}_{310})\varphi_{y}+R_{100}\varphi_{z}\right]/\varphi_{z};\;\nabla \varphi \in I\\ \left|\nabla \varphi \right|\left[3(R_{310}+{3R}_{311}-{4R}_{411})\varphi_{x}+3(R_{311}-R_{310})\varphi_{y}+({4R}_{411}-{3R}_{311})\varphi_{z}\right]/\varphi_{z};\;\nabla \varphi \in \text{II}\\ \left|\nabla \varphi \right|\left[6(R_{210}-R_{310})\varphi_{x}+3(R_{311}-R_{310})\varphi_{y}+({3R}_{310}-{2R}_{210})\varphi_{z}\right]/\varphi_{z};\;\nabla \varphi \in \text{III}\\ \left|\nabla \varphi \right|\left[10(R_{530}-R_{210})\varphi_{x}+({3R}_{311}-{8R}_{210}-{5R}_{530})\varphi_{y}+({6R}_{210}-{5R}_{530})\varphi_{z}\right]/\varphi_{z};\;\nabla \varphi \in \text{IV}\\ \left|\nabla \varphi \right|\left[5(R_{530}/3+{2R}_{211}/3-R_{311})\varphi_{x}+({8R}_{211}/3-{5R}_{530}/3-R_{311})\varphi_{y}+({3R}_{311}-{2R}_{211})\varphi_{z}\right]/\varphi_{z};\;\nabla \varphi \in V\\ \left|\nabla \varphi \right|\left[5({3R}_{530}+{2R}_{320})\varphi_{x}+({3R}_{320}+{2R}_{211}-{5R}_{530})\varphi_{y}+5({2R}_{530}-{3R}_{320})\varphi_{z}\right]/\varphi_{z};\;\nabla \varphi \in \text{VI}\\ \left|\nabla \varphi \right|\left[2({3R}_{221}-R_{211})\varphi_{x}+2(R_{221}+{2R}_{211}/3-R_{320})\varphi_{y}+(R_{320}-{4R}_{221}+{4R}_{211}/3)\varphi_{z}\right]/\varphi_{z};\;\nabla \varphi \in \text{VII}\\ \left|\nabla \varphi \right|\left[3({3R}_{331}-R_{320}-{2R}_{221})\varphi_{x}+6(R_{221}-R_{331})\varphi_{y}+({3R}_{320}-{6R}_{331}+{4R}_{221})\varphi_{z}\right]/\varphi_{z};\;\nabla \varphi \in \text{VIII}\\ \left|\nabla \varphi \right|\left[15(R_{540}-R_{320})\varphi_{x}/2+3({3R}_{320}/2-5R_{540}/2+R_{331})\varphi_{y}+({6R}_{320}-{5R}_{540})\varphi_{z}\right]/\varphi_{z};\;\nabla \varphi \in \text{IX}\\ \left|\nabla \varphi \right|\left[5(R_{110}-R_{540})\varphi_{x}+3(R_{331}-R_{110})\varphi_{y}+({5R}_{540}-{4R}_{110})\varphi_{z}\right]/\varphi_{z};\;\nabla \varphi \in X\\ \left|\nabla \varphi \right|\left[2(R_{221}-R_{211})\varphi_{x}+2(R_{111}-R_{221})\varphi_{y}+({2R}_{211}-R_{111})\varphi_{z}\right]/\varphi_{z};\;\nabla \varphi \in \text{XI} \end{array}$$

The necessary first derivatives can be derived directly from (18), but the corresponding expressions are too cumbersome to be stated here.

The second derivatives of the Hamiltonian appearing in (11) and (15) are also needed for checking their convexity condition (7). Actually, it is not necessary as the Figure 3 and 4 suggest that the etching rates (and corresponding Hamiltonians) are non-convex functions. It means that it is convenient to implement already mentioned procedure [25] in order to solve numerically initial value problem (5).

## 4. Simulation Results

Potassium hydroxide (KOH) is the most common and the most important chemical etchant, because of its excellent repeatability and uniformity in fabrication, and its low production cost. In actual calculations we made use of etching rates listed in Table 1. The actual shapes of the initial surfaces are described using simple geometrical abstractions. In the beginning of the calculations this descriptions are transformed into the initial level set functions using the fast marching method [15]. If the initial profile is defined with a bitmap mask, a special routine is used to generate corresponding initial level set function starting from the mask file. Our implementation is based on ITK library [24]. The classes describing the level set function and the level set filter are reimplemented according to the procedures for treating non-convex Hamiltonians described in the previous section.

Here we shall present some results obtained using previously described methodology. In view of the fact that we were not able neither to compare our results with cellular automata simulations nor to perform any experimental work, we have carefully chosen set of examples for which the outcomes (final profiles) are known, either from simple theoretical considerations or from published experimental results. For example, it is expected that the final shape after the etching of any 3D object must be composed of the fastest etching planes. Similarly, etching through the aperture of any form ends with a cavity bounded by the slowest {111} planes [1].

**Figure 5.**
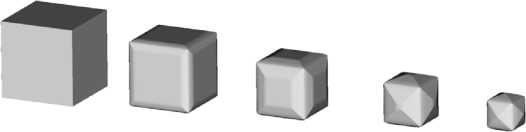
Etching profiles of the silicon cube with initial edge of 30 μm after 0 s, 120 s, 240 s, 360 s and 480 s, obtained using the thirteen-parameters interpolation formula for 30% etchant concentration.

Since the cube is the simplest isometric crystal form [26], first we present the time evolution of the initial cube shape made of {100} planes. In Figure 5, the changes of the cube form are shown at five equidistant time moments for 30% etchant concentration and thirteen-parameters interpolation formula.

It is obvious that the initial cube shape gradually transforms to the final tetrahexahedron, consisting of 24 triangles belonging to the {012} family of planes, through the combinations of these shapes. It is expected given that tetrahexahedron is the only isometric form made of {012} principal planes. If the fastest planes are not {012} family, as in the case of 40% etchant concentration, the final profile shape will change accordingly. We shall pay more attention to this in the next, more interesting case.

**Figure 6.**
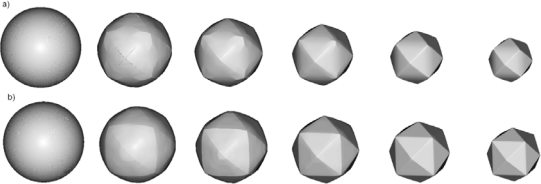
Etching profiles of the sphere with initial radius of 25 μm after 0 s, 100 s, 200 s, 300 s, 400 s, 500 s and 600 s, obtained using thirteen-parameters interpolation formulas for (a) 40% and (b) 50% etchant concentrations.

In order to test the strength of the method we have chosen to simulate etching of the silicon ball in KOH etchant. The initial spherical surface contains all possible velocity directions, so it is expected that the anisotropy of the etching process will produce the most dramatic changes of the initial shape. This shape, or more precisely hemisphere, is used in the experimental setup [17,27] for measuring etching rates anisotropy, also. In such an experiment a hemisphere is only etched for a short time in order to minimize the inteference of neighbouring orientations. Here we shall follow etching process until its final stage. Figure 6 the illustrates changes of the initial spherical shape at six equidistant (reduced) time moments for 40% and 50% etchant concentrations. The results are different from preliminary ones published in reference [19], where calculations with three parameters etching rates were performed on coarser mesh and with underestimated smoothing term in Lax-Friedrichs finite difference scheme. In the first case the final stage is (quasi) dodecahedron since the planes {110} are the fastest family. By word “quasi” we mean curved, since {110} planes forming dodecahedron are obviously bent toward {012} planes of the corresponding tetrahexahedron. The higher order fast etching planes ({320}, {530} and {540}) have no influence on the final shape, although some tetrahexadral forms probably can be recognized in the early phases of the profile evolution. This problem requires more careful analysis that goes beyond the scope of the present paper. For 50% etchant concentration (Figure 6b), the final shape is pure tetrahexahedron, since the fastest plane family is {012}, as can be seen from Table 1. The shape evolution for 30% etchant concentration is almost the same (a little bit faster), so it is not shown here.

**Figure 7.**
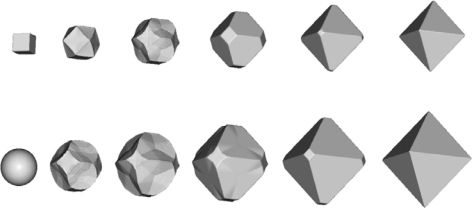
Artificial growth of the cube (upper row) and the sphere (lower row) with the growth rates given by the thirteen-parameters etching rate interpolation formulas for 30% etchant concentration.

In Figure 7 process inverse to etching, namely, the artificial growth of the cube and the sphere when growth rate is equal to the etching rate, is presented.

The final shapes are the same in both cases; it is octahedron made of the slowest {111} family of planes. It can be obtained [28] by the Legendre transform of etching rate angular distributions:

(20) $$W(N)=\underset{N\prime \cdot N>0}{\text{inf}}\left[\frac{R(N\prime )}{N\prime \cdot N}\right]$$

It can be easily shown that applying transform (20) on etching rates defined by (1), (3) and (4) lead to octahedrons. This example shows that possibilities for using the inverse modeling for design purposes are very limited, since the information about the initial shape is lost quickly. Actually, it is connected with the detailed knowledge of the etching rate anisotropy, as well as with the mask orientation, and probably models based only on relatively small number of parameters (like one presented in this paper) are not sufficient for this purpose.

**Figure 8.**
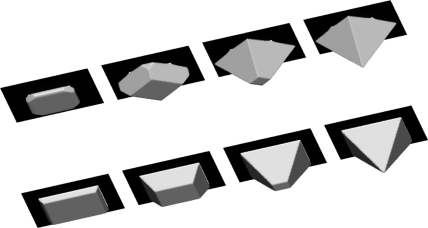
Etching through a square aperture mask in {100} plane aligned to <100> (upper row) and <110> (lower row) directions. Profiles after 100 s, 300 s, 600 s and 900 s, obtained using thirteen-parameter interpolation formulas.

The first example in which the maks are used is etching through square openings in the {100} silicon plane with edges aligned to <100> (Figure 8, upper raw) and <110> (Figure 8, lower row) directions. Formation of the V-shaped cavities consisting only of slow {111} planes is reproduced correctly in both cases. The masks square edge lengths are choosen to be $15\times \sqrt{2}$ μm and 15μm for <100> and <110> aligning directions respectively, so the final profiles are the same size cavities.

**Figure 9.**
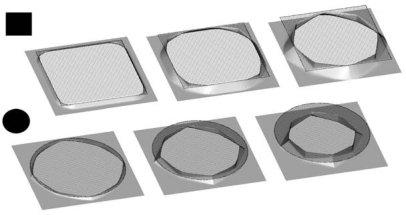
Convex corner undercutting. Initial aperture is aligned to <110> directions. Evolution of the profiles for the square (upper row) and the circular (lower row) masks, with the dotted masks superimposed, are presented.

Real applications very often require that mask includes, instead of only concave as in the previous case, many convex corners. In that case typical undercutting faceted shape beneath such a corner appears [29]. Convex corner undercutting can be a useful phenomena; for example it helps in the manufacturing of cantilever beam structures. On the other hand, it is highly undesirable in production mesa structures. Etching profiles evolution starting with two simple masks leading to pronounced undercutting phenomena are presented in Figure 9. Obtained profiles resemble, at least qualitatively, a SEM micrograph of an experimental mesa structure (see Figure 1 in reference [30]), although it seems to be more rounded then the experimental one. Generally speaking, convex corner undercutting is a very important and complex problem. Its proper modeling requires inclusion of a great number of high order planes [29], but we think that even relatively simple model with thirteen parameters describes it correctly. It is reasonable to assume that the inclusion of some other high order planes would enhance agreement with experimental shapes.

**Figure 10.**
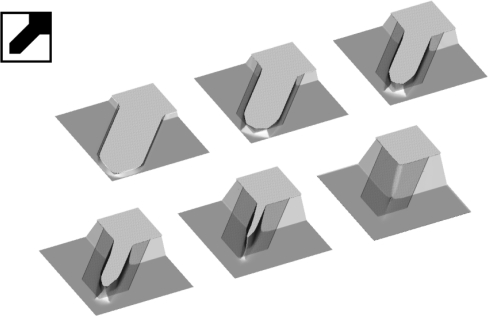
Convex corner compensation in mesa structure fabrication. Compensation mask is shown in the upper left corner. Initial aperture is aligned to <110> directions. Profiles at six equidistant time moments, with the dotted mask superimposed, are presented.

In order to avoid the effects of convex corner undercutting various compensation techniques are widely used. One of the most effective compensation structure is <100> oriented beam [29]. In Figure 10 the effects of such a compensation on the profile evolution during the mesa structure fabrication are shown.

**Figure 11.**
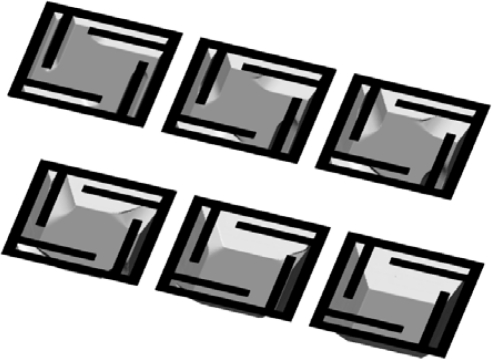
Formation of a system of suspended cantilever beams. Initial aperture is aligned to <110> directions.

**Figure 12.**
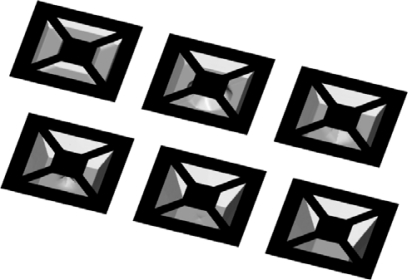
Formation of a released suspended plate. Initial aperture is aligned to <110> directions.

As it is mentioned in section 2, the anisotropic etching of sacrificial layers is usually used for manufacturing of suspended structures using. In Figures 11 and 12 evolutions of the etching profiles leading to formation of two different released structures are shown. The mask in Figure 11 contains both concave and convex corners, while that in Figure 12 includes only concave ones. In both cases the final profile is V-shaped {111} cavity beneath the suspended structure. Such structures are used for different purposes. Various types of cantilever beams (Figure 11) are required in accelerometers, light modulators, *etc.*

## 5. Conclusions

In this paper we have shown that the profile evolution during anisotropic wet etching of silicon can be described by the non-convex Hamiltonian arising in the Hamilton-Jacobi equation for the level set function. Angular dependence of the etching rate is calculated on the base of full silicon symmetry properties, by means of the interpolation technique using experimentally obtained values of the etching rates for principal and some of high order planes (totally thirteen) in KOH solutions. The resulting level set equations are solved by applying the sparse field method extended for the case of non-convex Hamiltonians. The simulation results showing profile evolution in some interesting 3D cases are presented: cube and sphere for various etchant concentrations. Also, examples are given showing that the method can be used to model etching process in the presence of masks. These include simple square aperture masks differently oriented, convex corner undercutting and convex corner compensation, formation of suspended structures. The results obtained so far show that level set method can be used as an effective tool for wet etching process modeling on the device level, and that it is a viable alternative to the cellular automata method which now prevails in the simulations of the wet etching process both in microscopic (atomistic) and engineering applications. However, much work is still needed to improve it to the level of the current commercial cellular automata simulators (for example, IntelliEtch [31]). It is especially important to reduce memory consumption which is, in our implementation, proportional to N^3^ (N-resolution in one spatial direction). We believe that it is also possible to use the level set method to analyze microscopic etching mechanisms on atomistic level, especially in conjunction with kinematic wave theory [32], and this will be a part of our future research.

## Figures and Tables

**Table 1.** Etching rates of silicon at different KOH concentrations at 70 °C [17,18].

**Crystallographic Orientation**	**30%**	**40%**	**50%**

(100)	0.797	0.599	0.539
(110)	1.455	1.294	0.870
(210)	1.561	1.233	0.959
(211)	1.319	0.950	0.621
(221)	0.714	0.544	0.322
(310)	1.456	1.088	0.757
(311)	1.436	1.067	0.746
(320)	1.543	1.287	1.013
(331)	1.160	0.800	0.489
(530)	1.556	1.280	1.033
(540)	1.512	1.287	0.914
(111)	0.005	0.009	0.009
(411)	1.340	0.910	0.660
